# Ex vivo demonstration of canine corneal pre-Descemet’s anatomy using pneumodissection as for the big bubble technique for deep anterior lamellar keratoplasty

**DOI:** 10.1038/s41598-022-24438-5

**Published:** 2023-04-11

**Authors:** Christiane Kafarnik, Lana A. Faraj, Darren S. J. Ting, Jia Ni Goh, Dalia G. Said, Harminder S. Dua

**Affiliations:** 1 Ophthalmology Unit, Animal Health Trust, Kentford, UK; 2 Academic Ophthalmology, Eye ENT Centre, School of Medicine, University of Nottingham, B Floor, Nottingham, NG7 2UH UK; 3 Department of Ophthalmology, Queen’s Medical Centre, Nottingham University Hospitals NHS Trust, Nottingham, UK; 4 School of Veterinary Medicine and Science, University of Nottingham, Nottingham, UK

**Keywords:** Animal physiology, Anatomy, Corneal diseases

## Abstract

The recent discovery and characterization of pre-Descemet’s layer (PDL; also termed the Dua’s layer or the Dua-Fine layer) has advanced the understanding of various posterior corneal pathologies and surgeries in human. This study aimed to characterize the ultrastructure of the posterior stroma and interfacial zone of Descemet’s membrane (DM) in canine eyes. Eighteen canine corneo-scleral discs were included. Intrastromal air injection resulted in the formation of type 1 big bubble (BB) in 73% (n = 11/15) of corneas, with a mean diameter of 11.0 ± 1.3 mm. No type 2 BB was created. Anterior segment optical coherence tomography, histology and transmission electron microscopy confirmed that the wall of BB was composed of DM, in contact with remaining stroma (canine PDL; cPDL). The cPDL was populated with keratocytes, of varying thickness of 16.2 ± 4.2 µm in close apposition to the DM, and composed of collagen bundles arranged in transverse, longitudinal and oblique directions. The interfacial zone, between DM and cPDL, showed fibril extension in all three directions, predominantly longitudinal. Irregular extensions of DM material into cPDL stroma were observed. No long-spaced collagen was detected. In conclusion, there exists a well-defined cleavage plane between the posterior stroma and cPDL, with similar but not identical characteristics as in humans, that is revealed by pneumodissection. This adds to our understanding of the anatomy of the posterior most canine cornea, which will have significant clinical impact on posterior corneal surgery and understanding of corneal pathology in dogs.

## Introduction

The canine cornea is a highly organized nearly transparent tissue forming the anterior part of the fibrous tunic of the eye and is an essential part of the optical system. The basic anatomic structure of the cornea among mammals is similar, with an anterior epithelium and its basement membrane, a broad stroma, Descemet’s membrane (DM), the basement membrane formed by the single layered posterior endothelium. However, a distinct layer comparable to the anterior limiting lamina (Bowman’s layer; BL) as in humans does not exist in dogs^1,2^. The substantia propria/stroma in dogs comprises approximately 90% of the entire thickness of the cornea. The density of keratocytes decreases from anterior stroma towards posterior stroma^3^. An ultrastructural analysis showed that the central cornea has significant smaller fibril diameter than the peripheral cornea in healthy beagle dogs. The narrower central fibrils are believed to buffer the direct impact of the intraocular pressure and the larger diameter peripheral fibrils allow high-density intermolecular crosslinks to provide tensile strength as the cornea merges with the sclera^4^.

The human interface between DM and pre-Descemet’s layer (Dua’s layer, PDL) is composed of a transitional zone of amorphous extracellular matrix which is formed by 2 years of age^5^. DM is composed of collagen and non-collagenous components^5–8^. On electron microscopy, two zones were described: a fetal, banded zone adjacent to corneal stroma, composed of irregularly patterned mainly type IV collagen, and a postnatal, posterior non-banded zone composed of homogenous, fibro-granular material^5^.

There is a growing interest in corneal anatomy and ultrastructure of the dog cornea as advances in human corneal surgeries are extrapolated to veterinary surgery. Deep anterior lamellar keratoplasty (DALK) procedure in veterinary ophthalmology is gaining popularity, but peer-reviewed publications in dogs are limited^9–11^. DALK is advantageous as it preserves the host corneal endothelium and DM. This method is increasingly becoming the method of choice due to decreased risk of graft rejection, preservation of the globe integrity, and reduced endothelial cell loss^12–14^. Technically DALK is challenging as it requires the dissection of the DM from the posterior corneal stroma. The most popular method described for this dissection is the Anwar’s big bubble (BB) technique (pneumodissection)^15,16^, which involves injection of air into the corneal stroma to separate DM. For many years, the exact cleavage plane reached with this technique was thought to be the interface between the posterior stroma and DM. In 2013, Dua et al^17^. discovered in a series of experiments that in 80–85% of samples the cleavage plane was between the deep stroma on one side and the well-defined PDL and the DM on the other. This type of BB was called a type 1 BB. The type 2 BB is less common and forms when air accesses the cleavage plane is between the PDL and the DM. A mixed BB, which is a combination of both type 1 and type 2 can also occur, wherein the type 2 component can be partial or complete^17^. The PDL in the human cornea is a 10.2 ± 3.6 µm thick layer, composed of 5–8 thin lamellae of longitudinal, transverse, oblique collagen bundles. PDL is described as impervious to air, of high tensile strength, mainly composed of collagen I, with high content of elastin and elastin-like fibers and a paucity of keratocytes^18,19^. The knowledge of the microstructure of the posterior stroma made the DALK procedure safer in humans and because of the diameter of a type 1 BB, a maximal trephine diameter of 8.5 mm is therefore recommended^18^. In addition, the discovery and characterization of PDL has also helped improve the understanding and management of various corneal pathologies, including acute hydrops^20,21^ and DM detachment^22^.

Currently, there is no information available about the microanatomy of the posterior stroma and the relation to the DM in dogs. The aim of this study was to characterize the microarchitecture of the posterior canine cornea using the BB technique and to determine the presence of canine PDL (cPDL).

## Results

A total of 18 canine corneo-scleral discs from 9 medium/large breed dogs were included in this study. The mean age (± standard deviation; SD) was 3.7 ± 2.2 years (range: 1–9 years), which was equivalent to approximately 30 years of human age (range: 15–56 years)^23^.

**Figure 1:**
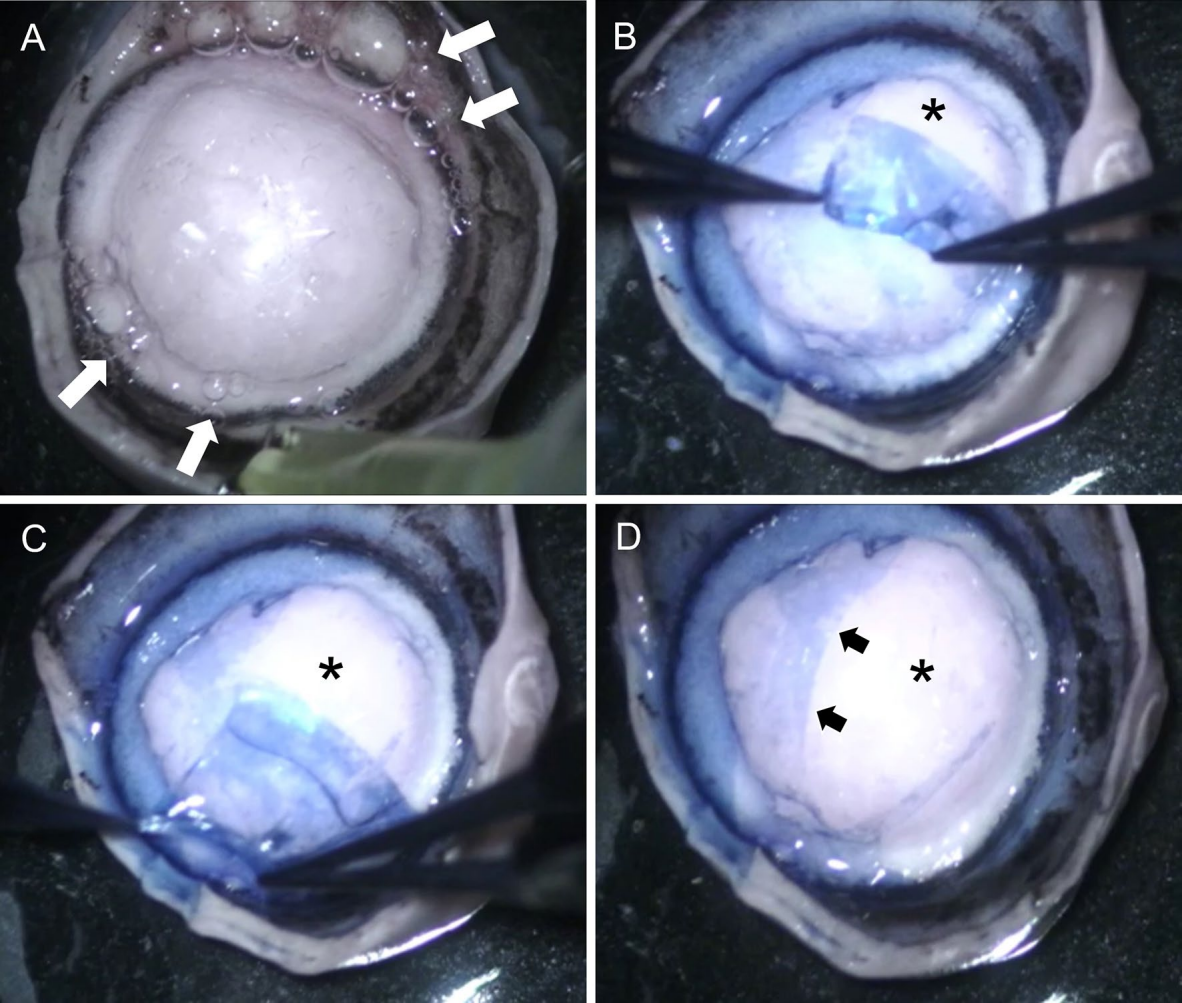
Macroscopic photographs of a type 1 big bubble (BB) in canine cornea after pneumodissection (corneal endothelium facing up). (**A**) A large central type 1 BB is present. White arrows point to the bubbles of air escaping from the periphery along the pigmented tissue. These bubbles are likely to enter the anterior chamber during big bubble deep anterior lamellar keratoplasty. (**B**) The Descemet’s membrane (DM) is being peeled off the BB revealing the posterior surface of the canine pre-Descemet’s layer (cPDL) (star). (**C**) On further peeling the DM tears as a broad strip, revealing more of the cPDL (star). (**D**) A broad central strip of the DM is peeled off. The edge of the remaining DM is clearly seen (black arrows). The DM was stained with vision blue dye (**B**–**D**).

The corneal diameter (limbus to limbus, anteriorly) ranged between 15 and 17 mm. A type 1 BB was obtained in 73.3% (n = 11/15) corneo-scleral discs. In three corneo-scleral discs, the needle had perforated through full thickness of cornea and had to be discarded. In one corneo-scleral disc, there was excessive leakage of air at the periphery, which was cut too close to the cornea and a BB could not be created. In all 11 remaining corneas, a type 1 BB was obtained. The type 1 BB was a well circumscribed, central dome-shaped elevation with a mean diameter of 11.0 ± 1.3 mm (range: 9.0–12.0 mm). The injected air travelled from the site of injection, backwards along the needle towards the limbus and then circumferentially along the peripheral cornea in clockwise and counterclockwise directions to complete the circle and then fill the central stroma (Videos 1 and 2). Continued injection of air resulted in several small bubbles forming in the center, which coalesced into one bubble, which enlarged and expanded towards the periphery and antero-posteriorly. While creating the BB, it was noted that tiny bubbles of air leaked from random points at the peripheral cornea, close to the inner pigmented band along a narrow circumferential strip of trabecular meshwork tissue (Videos 1, 2 and Fig. 1A).

Once a BB was formed, the DM could be peeled from the posterior (convex) surface of the BB as broad strips but not as a complete sheet. In none of these samples did the BB collapse on peeling the DM (Fig. 1B–D and Video 3). The air injected to create a BB ranged was between 15 and 20 ml. Attempts to rupture the once formed BB by forceful injection of more air (i.e., > 60 ml) failed as the air leaked through the peripheral cornea just inside the pigmented line. After retracting the needle, the BB was stable and maintained its configuration until further processing.

**Figure 2:**
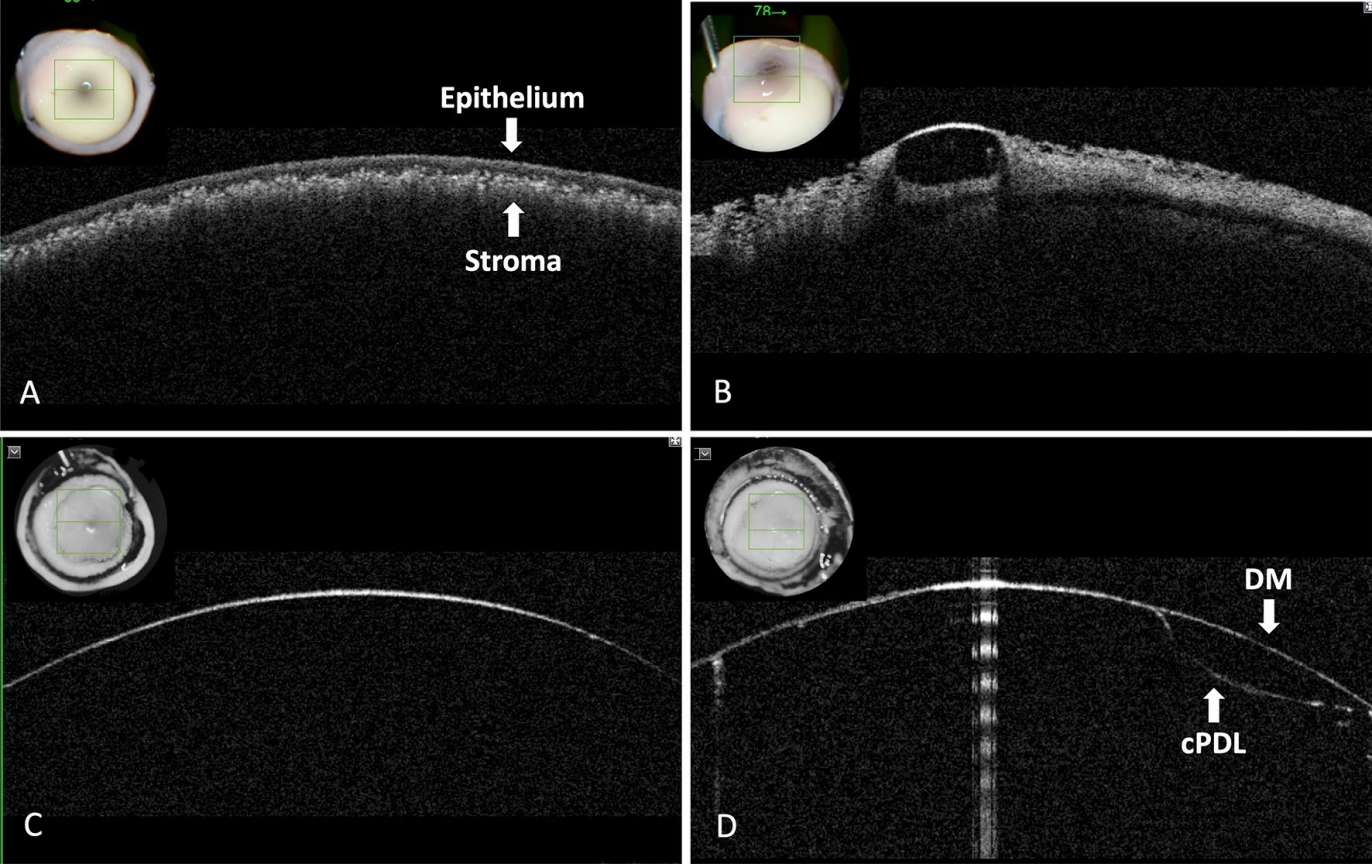
Anterior segment optical coherence tomography (AS-OCT) (Topcon Europe medical BV, Netherland) of the canine corneas after intrastromal air injection. (**A**) Anterior scan from the epithelial surface. The epithelium is demarcated from the underlying hyper-reflective aerated stroma. (**B**) Air has entered the epithelium creating and air vesicle. (**C**) Scan performed from the posterior surface of the cornea after creating a type 1 big bubble (BB). The posterior wall of the type 1 BB made of the canine pre-Descemet’s layer (cPDL) and Descemet’s membrane (DM). The AS-OCT scan does not distinguish between the two layers. (**D**) Similar scan as in ‘C’ of a type 1 BB. On the right side, the DM was peeled off over a section of the bubble and replaced prior to the scan. The anterior DM and posterior cPDL are clearly visible but are indistinguishable in the left two thirds of the scan where the DM and cPDL are closely apposed.

On anterior segment optical coherence tomography (AS-OCT), the BB layer was clearly imaged and identified, with a mean wall thickness of 47.6 ± 0.3 µm (ranged: 41.1–53.3 µm). In two specimens where the DM was peeled and replaced, an obvious two layered wall could be distinguished. The inner part of the BB wall corresponding to the cPDL, measured 18.2 ± 5.3 µm (Fig. 2).

### Light and transmission electron microscopy

**Figure 3:**
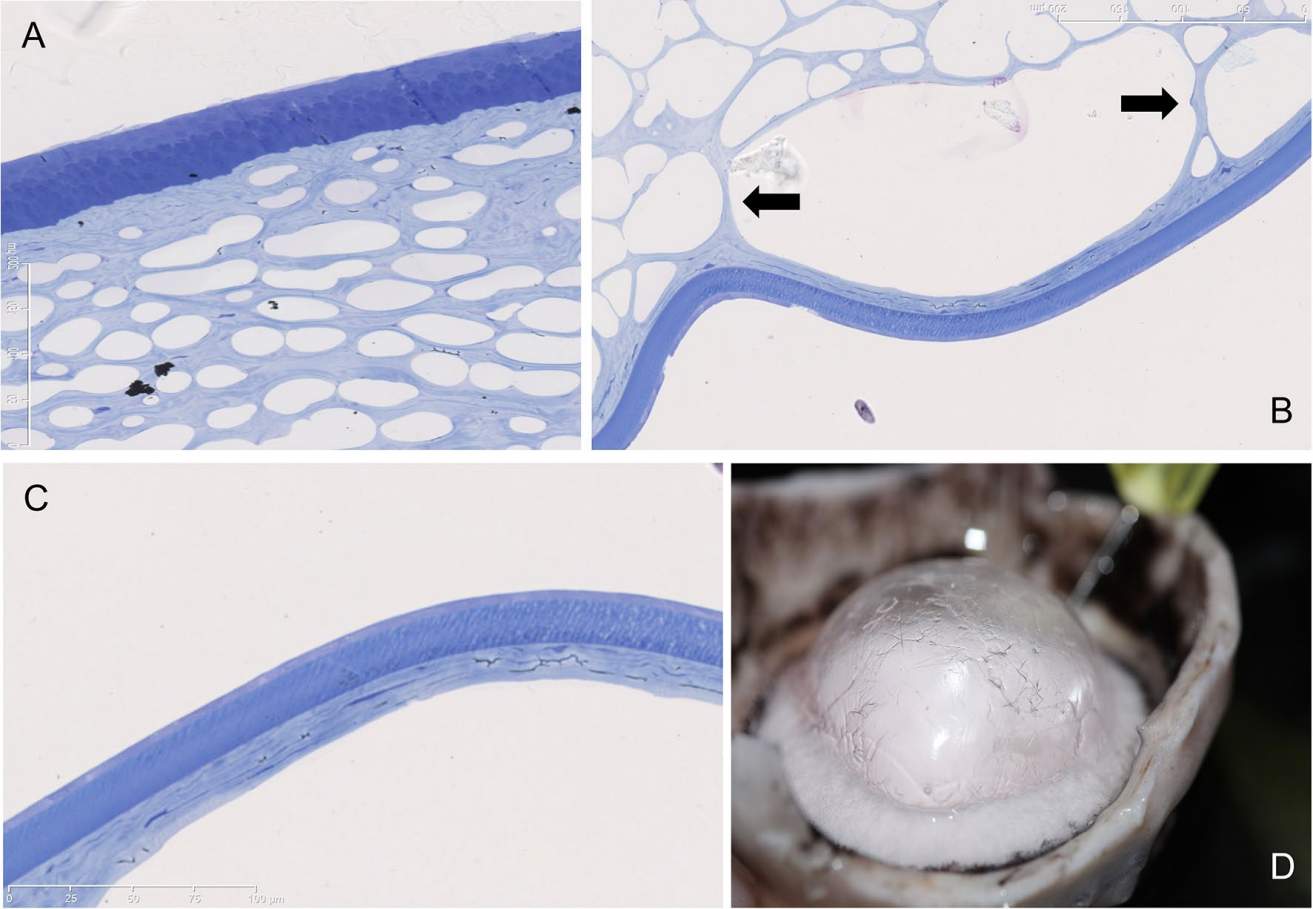
Histology of the canine cornea after air injection. (**A**) The anterior stroma and epithelium are illustrated. Air bubbles, larger posteriorly and smaller anteriorly are seen to fill the stroma. The air bubbles extend very close to the posterior surface of the epithelium. (**B**) The posterior stroma shows larger intrastromal bubbles. Arrows point to thin strands of collagen that extend between posterior stroma and the separating canine pre-Descemet’s layer (cPDL). (**C**) The separated cPDL (light blue) and the overlying Descemet’s membrane (DM) are illustrated. The cPDL shows horizontally aligned darker staining linear section of keratocytes. (**D**) The ‘rough’ appearing surface of the wall of a type 1 big bubble (BB) is illustrated. This appearance is related to the broken strands of collagen illustrated in ‘B’ above, that lie on the anterior surface of the cPDL within the BB. The histology sections are stained with toluidine blue (**A**–**C**).

**Figure 4:**
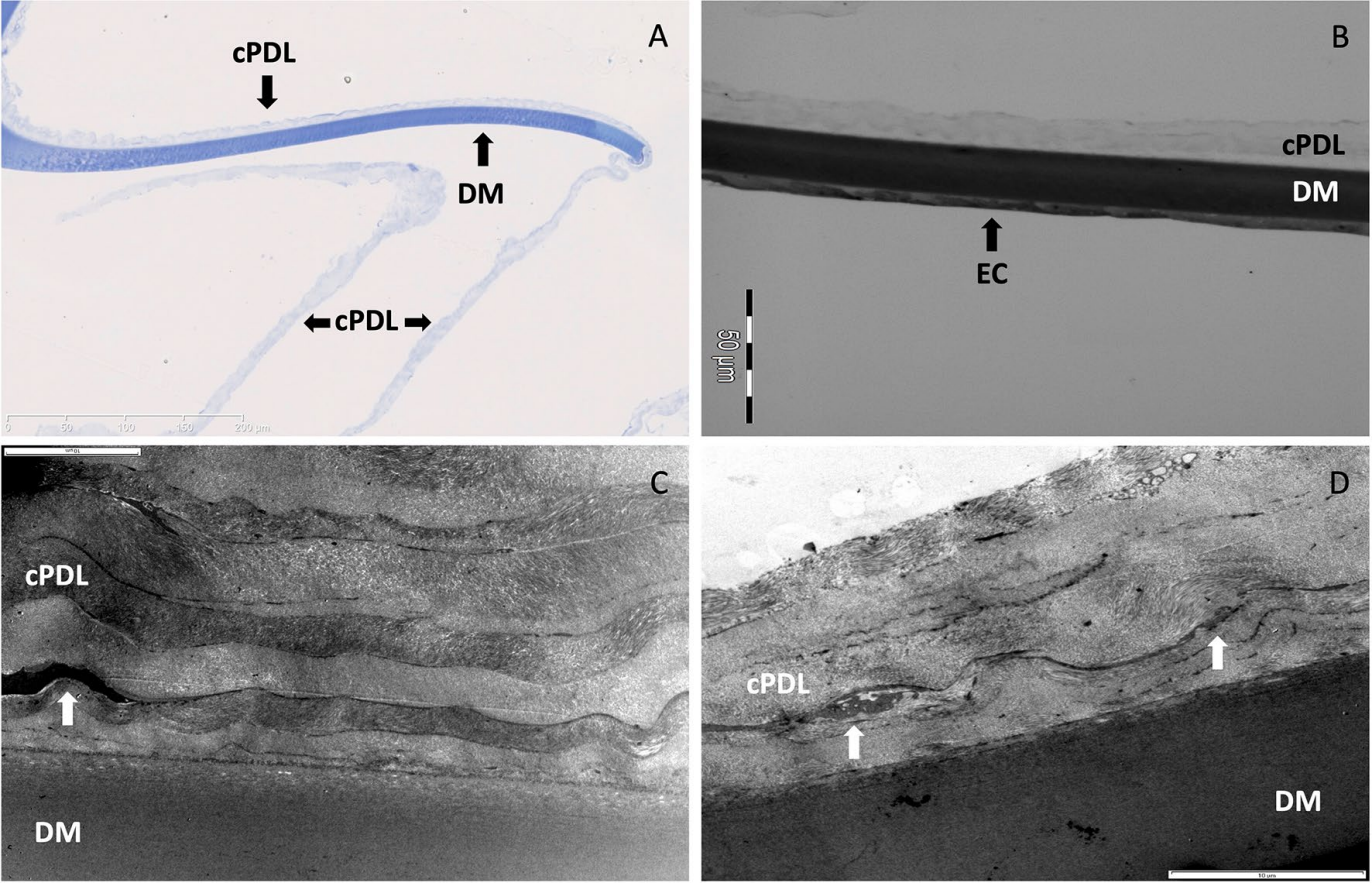
Histology of the canine pre-Descemet’s layer (cPDL). (**A**) Toluidine blue stained section of the cPDL and the Descemet’s membrane (DM). The DM is attached to part of the section of the cPDL. (Bar = 200 microns with 50-micron sections). (**B**) Transmission electron microscopy (TEM) of the wall of the type 1 big bubble (BB). The cPDL, DM and endothelial cells (EC) are illustrated. (Bar = 50 micros with 10-micron sections). (**C**) TEM of the cPDL anterior to the DM. A keratocyte is seen (white arrow). The compactly arranged horizontal and vertical lamellae are seen. Some oblique fibres are also visible (up and left) (Bar = 10 microns). (**D**) TEM of another section of cPDL, anterior to DM, showing keratocytes (white arrows) (Bar = 10 microns).

**Figure 5:**
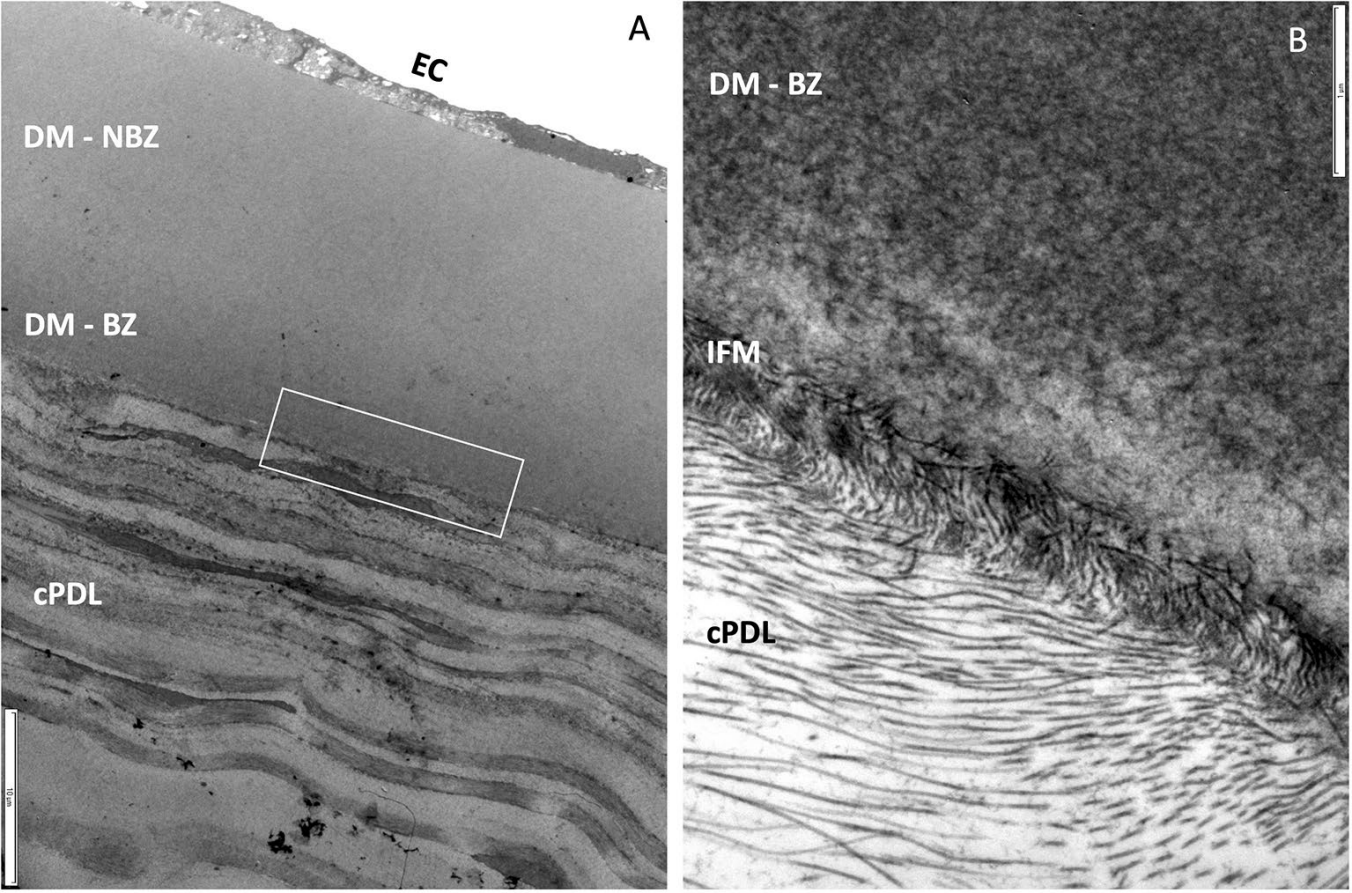
Histology of the interfacial matrix between the Descemet’s membrane (DM) and the canine pre-Descemet’s layer (cPDL). (**A**) Transmission electron microscopy (TEM) of the DM and the cPDL. The compacted alternating layers of the varying thickness of the cPDL are visible. (EC = endothelial cells. DM–NBZ = non-banded zone of the DM. DM–BZ = banded zone of the DM). The boxed area with the interfacial matrix is illustrated in high magnification in ‘B’. (Bar = 10 microns). (**B**) The interface between the DM and the cPDL is demonstrated. Fine fibrils are seen extending between the anterior surface of the PDL and the posterior surface of the banded zone of the DM (DM–BZ). Such fibrils are visible through most of the interfacial matrix (IFM) (Bar = 1 micron).

Histologically, the posterior wall of the BB (type 1) was composed of cPDL and DM. The cPDL was seen anterior to the DM, along the entire surface of the wall of the BB. The entire stroma of the cornea from which the BB had separated was filled with air spaces (Fig. 3A,B). Keratocytes were seen in and on the surface of the cPDL (Fig. 3C). Strands of collagen that extend from the deep stroma to the anterior surface of the cPDL and break to rest on the cPDL, giving the type 1 BB a ‘rough’ appearance (Fig. 3B,D), could be clearly seen. The mean cPDL thickness was 16.2 ± 4.2 µm (range: 7.5–21.6 µm). The mean DM thickness measured 21.5 ± 1.4 µm (range: 19.8–24.0 µm). The PDL was composed of loosely arranged collagen lamellae, consisting of longitudinal, vertical and oblique oriented fibrils. Keratocytes in the cPDL were seen in all samples at a mean distance of 1.4–12.4 µm from the anterior surface of DM but never in immediate contact with the DM (Fig. 4). In air injected corneas, the interfacial zone showed a dense network of fibrils penetrating into DM (0.5 µm) in different directions (Fig. 5). In the uninflated control corneas, the mean DM width measured 22.6 ± 3.1 µm (range: 18.8–28.4 µm). A clearly defined junction between the banded and non-banded zones could not be seen. The mean number of stromal collagen lamellae in the posterior stroma (up to 20 µm into the posterior stroma) measured 10.5 ± 3.6 (ranged: 7–16) collagen lamellae, and the mean thickness of individual stromal bundle measured 2.2 ± 1.3 µm (range: 0.3–6.5 µm).

The mean fibril diameter close to the DM in the area of the cPDL measured 27.0 ± 6.4 nm (range: 10.2–48.5 nm), which was smaller but not statistically significant than in the mid-stroma (29.0 ± 5.6 nm (range: 10.1–69.8 nm). Long spacing collagen was not present in any of the samples. Morphometric data is summarized in Table 1.

**Table 1:** Morphometric data of the canine pre-Descemet’s layer and the Descemet’s membrane.

Air injected cornea	Mean ± SD	Range
BB type 1 (n = 11/15)	11.0 ± 1.3 mm	9.0–12.0 mm
cPDL	16.2 ± 4.2 µm	7.5–21.6 µm
DM	21.5 ± 1.4 µm	19.8–24.0 µm
Keratocytes in cPDL (distance to DM)	–	1.4–12.4 µm
**Controls**		
DM	22.6 ± 3.1 µm	18.8–28.4 µm
Collagen lamellae number (up to 20 µm in stroma)	10.5 ± 3.6	7–16
Collagen lamellae thickness (up to 20 µm in stroma)	2.2 ± 1.3 µm	0.3–6.5 µm
Fibril diameter in cPDL zone	27.0 ± 6.4 nm	10.2–48.5 nm
Fibril diameter in mid-stroma	29.0 ± 5.6 nm	10.1–69.8 nm

*SD* standard deviation, *BB* big bubble, *cPDL* canine pre-Descemet’s layer, *DM* Descemet’s membrane.

## Discussion

The human PDL was discovered from observations made intraoperatively during DALK surgery by the BB technique, when certain clues suggested that the DALK by pneumodissection was not a DM baring technique as was hitherto believed^14,18^. The observations were subsequently confirmed by ex vivo experiments on human eye bank eyes, which clearly demonstrated the three types of BB, highlighting two distinct cleavage planes, one between the deep stroma and the PDL and the other between the PDL and the DM. The PDL, is impervious to air as the type 1 BB did not deflate on peeling of the DM. Moreover, a bubble between the posterior stroma and PDL could be created in corneas from which the DM was removed before air injection^18^. Laser ablation (or phototherapeutic keratectomy) of the PDL prior to injection of air failed to create a bubble^24^. Type 2 BB and the type 2 component of mixed BB were made by accumulation of air between PDL and DM and were very vulnerable to rupture during surgery. Knowledge of the bubbles allowed better understanding of DALK surgery and enabled precautions to be taken to complete DALK successfully without rupture of the DM. With the adoption of lamellar keratoplasty in veterinary surgery and to ascertain whether the PDL is present in other species, the question was posed whether it exists in dogs and behaves in a similar way.

This study was conducted to address these two issues. The experiments have conclusively demonstrated that cPDL, together with the DM, separated from the stroma after air injection is present, consistently producing a type 1 BB and demonstrating the presence of a cleavage plane. The type 1 BB occurred in a similar manner to that seen in humans with radial, circumferential and centripetal migration of air, in that order, with the formation of a central BB^25^. The size of the BB was larger than what is seen in humans corresponding to the larger corneal diameter of the canine eyes tested^25^. Unlike human eyes, where type 2 and mixed BBs occur in around 15% of cases, none occurred in canine eyes. The type 2 BB in human eyes has been shown to be due to the passage of air, through tiny fenestrations in the PDL at the periphery where its fibres open out to form the core of the trabecular meshwork. It is also known that the attachment of the DM to the posterior surface of the PDL by the interfacial matrix in humans, is weaker or looser in old eyes where separation of DM is relatively easier. The canine corneas were younger and the attachment between DM and cPDL was found to be very strong as the DM could not be peeled off from a type 1 BB in a complete sheet as demonstrated in older (> 70 years) human corneas. The peripheral microanatomy of the canine cornea with reference to the existence of fenestrations would need to be explored in detail to understand why type 2 and mixed BB did not form. When broad strips of DM were peeled off the type 1 BB in canine eyes to reveal the underlying cPDL, in one case half the area of the cPDL, the BB remained intact confirming that like in human eyes, the cPDL is impervious to air. More work needs to be done on the microanatomy of the canine eyes with regard to the association of the cPDL with the canine trabecular meshwork, as has been established in the human eye^26^.

These results have surgical implications in that it supports the notion that DALK in canine eyes is largely associated with a type 1 BB, which is the more desirable and common event. They also suggest that a type 2 or mixed BB is unlikely for the age of the cohort of animals studied, however a larger number of canine eyes are required to further substantiate this observation. Corneas from older animals of one breed group (i.e. medium or > 10 years) will also need to be studied to establish any difference that could exist. The strong attachment of the DM to the cPDL, observed in this study, would indicate that stripping of the recipient DM during an endothelial keratoplasty, including Descemet stripping endothelial keratoplasty (DSEK) and Descemet’s membrane endothelial keratoplasty (DMEK), may be challenging, though this may be easier when the DM/endothelium is diseased and requires replacement.

Histological features studied by light microscopy and TEM of the canine corneas showed some similarities with human eyes but there are also some key differences. The stromal lamellae separate as air traverses through the stroma. The extent of separation provides information on the arrangement of the lamellae and the compactness of their arrangement^25^. The canine stroma showed smaller air spaces anteriorly and posteriorly near the cPDL whereas the central stroma showed larger spaces. This was consistent with what was seen in human corneal stroma^25^. The intrastromal air spaces were seen to extend closer to the epithelium than in humans, probably related to the absence of the Bowman’s layer and relatively less compact anterior stroma.

The cPDL lamellae had similar characteristics to human PDL in that they were relatively more densely arranged compared to the posterior stroma and had smaller fibrillar diameter although not statistically significant. cPDL however contained more keratocytes, which indicates that there is a well delineated structural layer after pneumodissection but not a defined anatomic layer, as shown in control TEM images. The cPDL also showed a variable range of thickness, which could be related to the amount of stroma that separates from the posterior stoma as ‘strands of collagen’ that remain attached to the separating cPDL/DM^18^. The age difference between the canine and human samples studied and reported thus far could also contribute to the differences observed.

The canine cornea is slightly oval shaped, and its dimensions range between 15.5 and 20 mm with a middle-sized dog typically ranging between 16 and 18 mm. The curvature varies between 8.5 and 11.4 mm with a medium-sized dog normally ranging between 8.5 and 9 mm radius^27,28^. Large breed dogs were shown to have slightly flatter corneas, therefore larger radius than small- or medium-sized dogs^27^. We measured 15–17 mm corneal diameter in the present study which is in the normal range for a medium-sized dog as involved in the study.

In an ultrastructural analysis, Nagayasu et al^4^. showed the central cornea to have significantly smaller (29.1 ± 3.8 nm) fibril diameter than the peripheral cornea (32.6 ± 3.5 nm) in healthy beagle dogs. The amount of decorin and lumican, the two major proteoglycans of the corneal substantia propria, were found to be larger in the central than at the peripheral cornea. Both proteoglycans have been shown to inhibit an increase in collagen fibril diameter and regulate fibrillary spacing. Small fibrils centrally may also buffer the direct impact of intraocular pressure whereas larger diameter fibrils have high-density intermolecular crosslinks to provide a strong resistance to tensile force of the peripheral cornea as it continues with the sclera^4^. We did not differentiate between peripheral and central cornea but had similar results of the central measurements. Interestingly the fibril diameter close to the DM were found to be smaller in contrast to the mid-stroma in our study, which was similar to that observed in humans (21.7 ± 2.4 nm in PDL and 24.2 ± 2.7 nm in corneal stroma). The overall fibril diameter in dogs seems larger than in humans^17^.

The human interface between DM and PDL is composed of an approximately 0.9 µm transitional zone of amorphous extracellular matrix (inter fascial matrix), which is formed by 2 years of age^5^. Long spacing collagen and randomly arranged fibrils are seen close to DM and single stromal collagen fibers (30 nm) penetrate DM (0.5 µm depth)^29,30^. This is in contrast to what we observed in dogs in the present study. Long spacing collagen could not be found in any of the samples, more discreet longitudinal fibrils were noted as were fibrils entering the DM in varying directions. Interestingly, an irregular formed inter-digitation of basement membrane material of the DM was seen extending in the cPDL, which is different to humans and might contribute to and explain the strong attachment of the DM to the cPDL. Presence of long spacing collagen is also associated with older age and could be a factor for its absence in the young canine corneas studied.

The DM is an exaggerated basement membrane of the corneal endothelium^5–8^. Electron microscopically, two zones were described: a fetal, banded zone adjacent to corneal stroma, composed of irregularly patterned mainly type I collagen, and a postnatal, posterior non-banded zone composed of homogenous, fibrogranular material. Observations in mammals suggest that only the posterior non-banded zone is synthesized continuously throughout adult life and the DM increases in thickness with age^31–33^. DM has a thickness of approximately 10–15 µm in adult dogs^6^. In the present study, the mean thickness of DM varied around 21–22 µm in the air inflated group as well as in the control group. Moreover, the dogs in our study were all of young age where a thinner DM was expected. Postmortem changes may explain the difference or a variation in changes in thickness according to species.

Future directions of this work would include immunohistochemical investigation of proteoglycans (keratocan, lumican and decorin) and collagen compositions that will add further insights. There was a controversy on how to define a PDL given the anatomic definition of a “layer”^34^. The current study confirms the absence of a demarcated anatomic layer in the canine control corneas without forcing the separation of corneal lamellae. However, the current study provides evidence of a strong interface between DM and the posterior stroma forming a structural layer when mechanically manipulated, defined as cPDL. Similar issues were voiced with regard to the human PDL when it was first reported. Substantial evidence has since emerged over the years, by both in vivo and ex vivo observations that support the presence of a ‘layer’. The PDL/cPDL has been demonstrated by ultrahigh resolution OCT in the living human corneas^35^ and by micro-optical OCT in the pig corneas, where it has been demonstrated as a distinct layer^36^. The PDL has been demonstrated as a defined layer by staining for elastin, both in sections of the intact human cornea and after cleavage induced by pneumodissection^19^. The spontaneous separation of the PDL along the cleavage plane in DM detachment^22^ and its clear role in acute hydrops in keratoconus^17,18,20,21,37^ have provided clinical evidence of the PDL and its importance. Similar studies and observations in dog corneas are required and are likely to follow, to support or refute the presence of the ‘layer’ and its implications.

Although DALK with BB technique has been previously described in canine corneas^11^, this study is the first to demonstrate the existence of the cPDL in dogs predominantly forming a type1 BB after pneumodissection. This is a significant finding as it adds to implications for understanding and refining lamellar corneal surgery techniques and understanding corneal pathology^20–22,38–40^. Follow-up studies with a larger sample size are required to further characterize the cPDL in a range of dogs, with varying breeds and sizes.

## Methods

This study was approved by the Animal Health Trust (AHT) ethic committee (39-2014). Corneo-scleral discs were harvested within 2 h post-mortem from privately owned canine cadavers donated for research purposes. The dogs had been humanely euthanized for reasons unrelated to this project with a lethal dose using commercial euthanasia agents Eutha 77 sodium (barbituric acid derivative, phenytoin sodium), Virbac AH Fort Worth, TX) via a cephalic venous catheter. All methods were performed in accordance with the relevant guidelines and regulations.

The cadaveric corneo-scleral discs were collected from both eyes from six Staffordshire bull terriers, one Boxer, one mixed-breed dog, and one Labrador retriever. Five dogs were male entire, two male castrated and two females spayed dogs. Three corneo-scleral discs from Staffordshire bull terriers were used as controls and the rest were used for air injection. The cadaveric eyes were examined by slit-lamp biomicroscopy, and the corneo-scleral discs were excised within four hours post euthanasia and maintained in a standard organ culture media at 4 °C with media changes every second day (Eagle’s minimum essential medium with 2% fetal bovine serum for a maximum of 14 days).

### Corneal air injection (big bubble technique)

Experiments were performed under an operating microscope and video documented. Air was injected in 15 corneo-scleral discs as described before^17^. Briefly, with the disc placed with the endothelial surface up, a 30 gauge needle, attached to a 10 ml syringe filled with air, was inserted approximately 2 mm from the limbus, advanced into mid-stroma for 5–6 mm towards the central cornea (Videos 1 and 2). Air was injected to produce a BB. In three specimens attempt was made to peel off the Descemet’s membrane from the BB and trypan blue ophthalmic solution (Acrivet blue, Bausch Lomb Inc., Berlin, Germany) was used for visualization (Fig. 1). The corneal diameter and BB diameter were measured using a pair of surgical calipers and the amount of air injected was documented.

### Anterior segment optical coherence tomography (AS-OCT)

AS-OCT was performed in seven BB specimens kept in saline within 24 h after the air injection using a Topcon 3D OCT-2000 (Topcon Europe medical BV, Netherland) with a depth resolution of 5 µm and a lateral resolution of < 20 µm. The BB wall thickness was measured in three locations per specimen using image J software (imagej.nih.gov/ij)^41^. In two of the three samples from which the DM was peeled to approximately half the width of the BB, the DM was replaced on the PDL prior to OCT examination, simulating a mixed BB.

### Histology

Three BB specimens and three control specimens were fixed in 10% buffered formaldehyde, paraffin embedded and 4 µm sections were stained with Toluidine blue using the standard procedure histological staining technique^17^.

### Transmission electron microscopy (TEM)

Three BB tissue samples and three corneo-scleral discs without air injection were prepared for TEM after a standard procedure^17^. TEM was carried out using a JOEL 1010 microscope (JOEL, Herts, UK).

### Controls

Three corneo-scleral discs without air injection were used as controls to compare the DM thickness, number and thickness of stromal collagen lamellae up to 20 µm from the DM in each of three locations per sample. The fibril diameter close to the DM and in the mid-stroma in three different locations per sample was measured using an automated morphometric software program provided by the manufacturer (JOEL, Herts, UK). Presence and location of keratocytes close to DM (< 20 µm) were counted. The connection of the interfacial zone between the DM and PDL was examined.

## Supplementary Information

Supplementary Video 1.

Supplementary Video 2.

Supplementary Video 3.

## Data Availability

The authors confirm that the data supporting the findings of this study are available within the article and its supplementary files.
